# Understanding Fibroadenoma of the Breast: A Comprehensive Review of Pre-operative and Post-operative Clinicopathological Correlations

**DOI:** 10.7759/cureus.51329

**Published:** 2023-12-30

**Authors:** Pranam Pandit, Siddhant P Murkey, Akash Agarwal, Arpita Jaiswal, Suyash Agrawal

**Affiliations:** 1 Medicine, Jawaharlal Nehru Medical College, Datta Meghe Institute of Higher Education and Research, Wardha, IND; 2 Medicine and Surgery, Jawaharlal Nehru Medical College, Datta Meghe Institute of Higher Education and Research, Wardha, IND; 3 Surgery, Jawaharlal Nehru Medical College, Datta Meghe Institute of Higher Education and Research, Wardha, IND; 4 Obstetrics and Gynaecology, Jawaharlal Nehru Medical College, Datta Meghe Institute of Higher Education and Research, Wardha, IND

**Keywords:** multidisciplinary collaboration, surgical management, diagnosis, benign, breast, fibroadenoma

## Abstract

Fibroadenomas of the breast are common benign lesions that predominantly affect young women. This review provides a comprehensive overview of fibroadenoma management, encompassing their definition, clinical presentation, diagnostic tools, surgical management, clinicopathological correlations, treatment outcomes, complications, and emerging research. Fibroadenomas typically present as palpable breast lumps, often with no associated nipple discharge, and their diagnosis relies on a combination of clinical examination, breast imaging, and pathological confirmation. Surgical interventions, including excisional biopsy and lumpectomy, offer symptom relief and favorable long-term outcomes. Minimally invasive techniques and ongoing research into genomics and molecular aspects hold promise for the future of fibroadenoma management. Multidisciplinary collaboration among healthcare providers is paramount, ensuring accurate diagnosis, personalized treatment decisions, and holistic patient care. As research advances, the management of fibroadenomas is poised to evolve, providing improved diagnostic accuracy, minimally invasive treatments, and enhanced patient outcomes.

## Introduction and background

Breast fibroadenomas, characterized by their rubbery texture and well-defined borders, are among the most common benign breast lesions encountered in clinical practice. These fibroepithelial tumors typically arise from the glandular and stromal tissues of the breast and primarily affect women in their reproductive years. While they are generally benign and pose a low risk of malignancy, fibroadenomas can engender a significant degree of concern and anxiety among patients due to their palpable nature. As such, a comprehensive understanding of fibroadenomas and their management is essential for clinicians and patients [1].

Breast fibroadenoma is a common benign breast condition that affects women, particularly in their 20s and 30s. The incidence and prevalence of fibroadenoma vary depending on the age group and population. The prevalence of breast fibroadenoma in women aged 18-40 years in a study conducted in Guangdong province of China was 27.6% [2]. The incidence of fibroadenoma decreases with age, and it is generally found before 30 years of age in females in the general population [1]. It is estimated that 10% of women have breast fibroadenomas [3]. Simple fibroadenomas have a reported incidence of 7-13% in women from adolescence through the mid-20s who present to specialty clinics [4]. In a tertiary care hospital study, fibroadenoma was found to be the most common benign breast disease, accounting for 55% of cases, with the majority of cases presenting between 20 and 24 years of age [4]. Age is a significant factor in the incidence of fibroadenoma, and a family history of breast cancer is also important. Female patients with first-degree relatives with breast cancer should be monitored and observed more carefully for malignant features than patients without this family history [4].

Managing fibroadenomas involves a critical decision-making process, particularly regarding whether surgical intervention is warranted. While fibroadenomas themselves do not pose a direct threat to a patient's life, the need for surgery arises from the need to definitively diagnose the lesion, alleviate patient anxiety, and rule out malignancy, which can occasionally mimic the clinical presentation of fibroadenomas. In this context, pre-operative and post-operative clinicopathological correlations are pivotal in ensuring optimal patient care [5].

Pre-operative clinicopathological correlations involve the integration of clinical findings, diagnostic imaging, and biopsy results to determine the necessity and extent of surgical intervention. Accurate pre-operative diagnosis and assessment enable healthcare providers to counsel patients effectively, select appropriate treatment modalities, and manage expectations [6]. Post-operative clinicopathological correlations are equally crucial as they provide insights into the nature of the excised lesion and can help validate or revise the initial diagnosis. These correlations are essential for confirming the benign nature of fibroadenomas, identifying any concurrent pathology, and assessing the completeness of the excision [7,8].

This comprehensive review aims to provide a thorough understanding of fibroadenoma of the breast, with a specific focus on the importance of pre-operative and post-operative clinicopathological correlations in its management. We aim to delve into the clinical, pathological, and surgical aspects of fibroadenomas, offering a comprehensive resource for healthcare professionals, researchers, and patients seeking clarity on this typical breast lesion.

## Review

Fibroadenoma: etiology and pathogenesis

Factors Contributing to Fibroadenoma Development

Several factors contribute to the development of fibroadenomas, reflecting the complex interplay of hormonal, genetic, and environmental influences. Firstly, hormonal influences, particularly estrogen and progesterone, play a pivotal role in the growth and maintenance of breast tissue. Fibroadenomas exhibit hormonal responsiveness, with their development or enlargement often coinciding with periods of hormonal fluctuations, such as puberty, pregnancy, and the menstrual cycle. The presence of hormone receptors in fibroadenoma cells underscores their sensitivity to hormonal cues, suggesting a driving force behind their growth [9]. Moreover, research indicates a genetic predisposition to fibroadenoma development, supported by the identification of specific genetic mutations or variations in some studies. For instance, it may be valuable to consider suggesting a particular mutation that has been associated with an increased susceptibility to fibroadenomas. Although the genetic basis of fibroadenomas remains intricate and not entirely understood, these findings underscore the complexity of the condition, suggesting that multiple genes and factors likely interact to influence their occurrence [10]. Reproductive factors are significant contributors to the risk of fibroadenoma development. Factors such as early onset of menstruation (menarche) and nulliparity (not having given birth) have been associated with an elevated likelihood of developing fibroadenomas. These reproductive factors are frequently linked to hormonal changes that contribute to the growth and development of these benign breast lesions [11]. Additionally, while trauma or injury to the breast tissue is not a direct cause of fibroadenomas, it may serve as a triggering event for their development or growth. Trauma can disrupt the standard tissue architecture within the breast, creating an environment conducive to the formation of fibroadenomas. It's noteworthy, however, that fibroadenomas can also develop spontaneously without any preceding injury, highlighting the multifaceted nature of their etiology [12].

Cellular and Histological Characteristics of Fibroadenomas

Fibroadenomas are biphasic, comprising two primary components: epithelial (glandular) and stromal (connective tissue) elements. The epithelial component forms gland-like structures resembling ducts and lobules, responsible for milk production. Meanwhile, the stromal component consists of spindle-shaped cells within a collagen-rich matrix, providing structural support and contributing to the firm texture of the lesion [13]. The predominant stromal cells are fibroblasts, specialized in producing collagen and other extracellular matrix components, defining the unique texture of fibroadenomas [14]. Histologically, fibroadenomas often display a lobulated or well-circumscribed appearance, with distinct lobules separated by fibrous tissue, a feature notable in imaging and surgical excision [1]. Despite standard histological features, fibroadenomas exhibit cellular variability, with some containing cystic spaces, calcifications, or hyalinized areas characterized by dense, pinkish tissue. These variations highlight the diverse histological presentations within the spectrum of fibroadenomas [1].

Risk Factors for Fibroadenoma

The occurrence of fibroadenomas follows a distinct pattern concerning age and gender, predominantly diagnosed in women aged 15 to 35 while being notably rare in postmenopausal women and exceedingly uncommon in men [1]. Hormonal factors play a pivotal role in the development and growth of fibroadenomas, with heightened risk periods coinciding with hormonal fluctuations during puberty, pregnancy, and the menstrual cycle. Women with hormonal imbalances or undergoing hormone replacement therapy face an elevated risk due to the responsiveness of fibroadenoma cells to estrogen and progesterone [6]. A familial history of fibroadenomas or other benign breast conditions can increase the likelihood of their development. Although the genetic mechanisms remain not fully elucidated, a familial predisposition to fibroadenomas has been observed, particularly in cases where close relatives have experienced these lesions [6]. Certain reproductive factors are associated with an increased risk of fibroadenomas, including early menarche and nulliparity, as hormonal changes during these reproductive phases contribute to favorable conditions for fibroadenoma growth [6]. Additionally, race and ethnicity seem to influence fibroadenoma prevalence, with some studies indicating a higher incidence among women of African descent. However, further research is required to comprehensively understand these variations and their underlying causes [6].

Clinical presentation

Signs and Symptoms of Fibroadenomas

Fibroadenomas are primarily characterized by a palpable breast lump, the most common and notable clinical feature. These lumps, usually painless, possess distinct characteristics, being well-defined, smooth, and endowed with a rubbery texture. While some individuals may experience mild discomfort or tenderness, the palpable mass is the primary impetus for seeking medical evaluation [1]. When fibroadenomas are sizable or multiple within the same breast, individuals may observe a discernible change in breast size or shape. This alteration typically occurs gradually, becoming more apparent during hormonal fluctuations associated with the menstrual cycle or pregnancy [1]. Another distinguishing feature is the mobility of the lump within the breast tissue, allowing it to be moved under the skin during palpation, a critical factor in distinguishing fibroadenomas from other breast lesions [1]. Fibroadenomas also exhibit variability in size, ranging from a few millimeters to several centimeters in diameter. While some remain stable, others may undergo growth, emphasizing the importance of monitoring size changes for appropriate management [1]. Notably, fibroadenomas typically do not produce nipple discharge, setting them apart from certain other breast conditions like intraductal papillomas. This absence of nipple discharge is a significant clinical clue in differentiating fibroadenomas from lesions with distinct characteristics [1].

Differential Diagnosis

Various breast conditions share clinical features with fibroadenomas, necessitating a careful consideration of differential diagnoses. Breast cysts, fluid-filled sacs within breast tissue resembling fibroadenomas, often exhibit size fluctuations and may cause breast pain. Detection through ultrasound imaging is standard, with fine-needle aspiration serving as a diagnostic confirmation [15]. Although presenting as palpable breast lumps like fibroadenomas, phyllodes tumors are distinct in histology, featuring leaf-like projections. Surgical excision is often recommended for larger phyllodes tumors [16]. Unlike fibroadenomas, breast cancer poses a potential concern, especially in older individuals or with specific alarming features. Imaging and biopsy are imperative for distinguishing between benign and malignant breast lesions [17]. Intraductal papillomas, benign growths within breast ducts causing nipple discharge, are typically non-palpable and detected through imaging modalities like mammography or ductography [18]. Trauma-induced fat necrosis, characterized by fat tissue cell death, may mimic a breast lump, often accompanied by pain and skin changes [19]. Fibrocystic breast changes, prevalent in reproductive-age women, can coexist with fibroadenomas, complicating clinical evaluation. Diagnostic imaging, such as ultrasound or mammography, aids in distinguishing fibrocystic changes from other lesions [20]. Careful consideration of these differential diagnoses is crucial for accurate diagnosis and appropriate management.

Importance of Early Detection

The identification of a breast lump often triggers considerable anxiety and concern. Early diagnosis of fibroadenomas plays a pivotal role in alleviating this anxiety by confirming the benign nature of the lesion. This knowledge provides reassurance and enables individuals to shift their focus toward appropriate management and care, minimizing the emotional impact [21]. Moreover, timely diagnosis helps avoid unnecessary surgical procedures or interventions, especially for small, asymptomatic fibroadenomas confirmed to be benign. By removing unnecessary interventions, individuals are spared from potential complications and surgery's associated physical and emotional toll, contributing to a more streamlined healthcare experience [22].

Early detection facilitates close monitoring in cases where fibroadenomas are more significant, symptomatic, or raise cosmetic concerns. Regular follow-up appointments and imaging studies enable healthcare providers to track changes in size or characteristics. This proactive approach is particularly crucial for identifying cases where surgical intervention may be necessary to address symptoms or cosmetic issues, leading to improved outcomes when intervention is initiated early during the condition [6].

Furthermore, early diagnosis ensures a thorough evaluation to rule out malignancy. While fibroadenomas are benign, breast cancer can present with similar symptoms, including palpable breast lumps. Early detection and appropriate diagnostic procedures, such as imaging and biopsies, are paramount for accurately differentiating between benign and malignant lesions. Early diagnosis of breast cancer significantly enhances the prospects of successful treatment and overall better outcomes, emphasizing the importance of prompt and precise diagnostic measures [1].

Diagnostic tools and imaging

Accurate diagnosis of fibroadenomas involves a combination of clinical assessment and imaging studies. Table 1 explores the various diagnostic tools and imaging modalities commonly used to evaluate fibroadenomas.

**Table 1:** Diagnostic tools and imaging for fibroadenomas

Diagnostic tool	Role
Clinical breast examination (CBE) [8]	A comprehensive evaluation conducted by a healthcare professional is integral to breast health. This involves a thorough examination to meticulously assess any detected lump's size, shape, texture, and mobility. Additionally, the healthcare provider examines the surrounding breast tissue, scrutinizing for abnormalities, skin changes, or nipple discharge. This meticulous examination is crucial for identifying palpable breast masses, including detecting and characterizing fibroadenomas.
Breast ultrasound [9]	An invaluable imaging tool in assessing breast lumps, including fibroadenomas, is ultrasound. This imaging modality provides detailed and high-resolution images of breast tissue, facilitating the characterization of the lump in size, shape, location, and internal structure. Particularly, ultrasound aids in differentiating fibroadenomas from other breast lesions, such as cysts, by highlighting distinctive features like a well-defined, oval or round shape, hypoechoic appearance, and smooth margins. Beyond diagnosis, ultrasound plays a crucial role in monitoring fibroadenoma growth over time through serial examinations. Moreover, it serves as a guiding force for biopsy procedures, ensuring precise tissue sampling and contributing to the overall accuracy of diagnostic interventions.
Mammography [10]	Mammography, a standard breast imaging technique, employs low-dose X-rays to identify breast abnormalities, including fibroadenomas. It plays a crucial role in identifying calcifications within fibroadenomas, contributing to the diagnostic process, although it's important to note that not all fibroadenomas exhibit calcifications. The effectiveness of mammography can be influenced by breast tissue density; dense tissue may pose challenges in differentiating fibroadenomas from other lesions or cancers. While mammography is essential for breast cancer screening and may incidentally detect fibroadenomas, additional evaluation through ultrasound or biopsy may be necessary for a conclusive diagnosis.
Biopsy techniques (fine-needle aspiration, core needle biopsy) [11]	Biopsy procedures are essential when imaging indicates the presence of a breast lump, serving to confirm the diagnosis and assess tissue for malignancy. Fine-needle aspiration (FNA) involves withdrawing cells or fluid from the lump using a thin needle. This method is suitable for cystic lesions or highly suggestive fibroadenomas, providing rapid results that aid in distinguishing between benign and malignant lesions. On the other hand, core needle biopsy (CNB) employs a larger, hollow-core needle to extract a tissue sample for thorough histological examination. CNB is recommended for solid lesions or situations where a definitive diagnosis is necessary, confirming the presence of fibroadenomas and ensuring a comprehensive understanding of the tissue characteristics.

Pre-operative assessment

The pre-operative assessment of fibroadenomas is a critical phase in the management of these breast lesions. It involves evaluating various aspects, including the necessity for surgical intervention, choosing the appropriate surgical approach, and ensuring that patients are well-informed and have consented to the procedure described in Table 2.

**Table 2:** Pre-operative assessment of fibroadenomas

Pre-operative Assessment	Description
Indications for surgical intervention [12]	Several factors may prompt the surgical removal of fibroadenomas. Surgical intervention may be deemed necessary if these noncancerous breast lumps induce pain, discomfort, or notable tenderness, warranting the need for symptom relief. Moreover, fibroadenomas exhibiting rapid or substantial growth can elicit concerns about potential malignancy, leading to surgical removal for a definitive diagnosis. The removal of large fibroadenomas or those altering breast size or shape may also be considered for cosmetic reasons or to address psychosocial distress. In cases of uncertain diagnosis or suspected breast pathology, surgical excision is recommended to obtain a comprehensive tissue sample for histological evaluation. Patient preferences and concerns play a significant role in the decision-making process, with some individuals opting for surgical removal based on their specific preferences. The indications for surgical excision in patients with fibroadenomas depend on various factors, and not all cases require immediate intervention. Small, asymptomatic fibroadenomas with a high degree of diagnostic certainty can often be managed through observation, involving regular clinical breast examinations and imaging to monitor changes over time. However, surgical excision becomes warranted in several scenarios. Firstly, if a fibroadenoma causes persistent pain, discomfort, or notable tenderness, surgical removal may be recommended to alleviate symptoms and enhance the patient's quality of life. Additionally, the rapid or substantial growth of a fibroadenoma may raise concerns about potential malignancy, prompting surgical removal for a definitive diagnosis. Cosmetic considerations, such as the presence of large fibroadenomas or those altering breast size or shape, may also serve as an indication for surgical intervention, especially if these factors significantly impact the patient's psychological well-being. In cases of uncertain diagnosis or suspected breast pathology, surgical excision is recommended to obtain a complete tissue sample for histological evaluation, ensuring an accurate diagnosis and ruling out any concurrent malignancy. Ultimately, the decision for surgical excision should be based on a thorough evaluation of the individual patient's clinical presentation, preferences, and the specific characteristics of the fibroadenoma.
Selection of the surgical approach (excision vs. observation) [13]	There are two primary approaches for managing fibroadenomas based on specific considerations. First, observation is deemed appropriate for small, asymptomatic fibroadenomas with a high degree of diagnostic certainty. This involves regular clinical breast examinations and imaging to monitor changes over time. On the other hand, surgical excision is recommended for fibroadenomas that meet specific indications. The surgical methods employed, such as lumpectomy or complete excision, depend on factors like the size and location of the fibroadenoma, as well as patient preferences.
Patient counseling and informed consent [12]	Communication and patient involvement are crucial in fibroadenoma management. Firstly, provide a comprehensive explanation of diagnosis and treatment options. Inform patients of benefits (symptom relief, diagnosis) and risks (scarring, infection). Discuss alternative approaches involving patients in shared decision-making based on their values. Obtain informed consent, ensuring that patients understand risks, benefits, and alternatives. Address psychosocial aspects of the diagnosis and treatment decision to provide holistic support.

Surgical management

Types of Surgical Procedures

Excisional biopsy: An excisional biopsy is a surgical procedure involving completely removing the fibroadenoma and a margin of healthy breast tissue. This approach is used when the diagnosis is uncertain or the patient prefers complete removal. It provides a tissue specimen that can be thoroughly examined histologically to confirm the presence of a fibroadenoma and rule out malignancy. Excisional biopsy may be performed under local or general anesthesia, depending on the patient's and surgeon's preferences [5].

Lumpectomy: A lumpectomy, also known as a partial mastectomy or breast-conserving surgery, is a procedure where only the fibroadenoma is removed while preserving the surrounding breast tissue. This approach is suitable for larger fibroadenomas or when preservation of breast appearance is a priority. A lumpectomy is often performed under general anesthesia. It is important to note that the term lumpectomy is more commonly associated with the treatment of breast cancer, but the same surgical principles can be applied to fibroadenoma removal [14].

Role of Minimally Invasive Techniques

Ultrasound-guided vacuum-assisted excision: It uses ultrasound guidance to locate and precisely remove the fibroadenoma. A small incision is made, and a vacuum-assisted device is used to suction out the fibroadenoma while sparing as much healthy breast tissue as possible. This approach is particularly suitable for smaller fibroadenomas and is performed under local anesthesia [15].

Radiofrequency ablation (RFA): RFA is a minimally invasive procedure that uses radiofrequency energy to heat and destroy the fibroadenoma tissue. It is performed under local anesthesia and typically involves a small incision through which an RFA probe is inserted. RFA may be a viable option for selected cases, but its long-term effectiveness and safety are still being studied [16].

Complications and Risks Associated with Surgery

Infection: Surgical site infections are a recognized risk of any surgical procedure, including fibroadenoma removal. Infections can manifest as localized pain, redness, swelling, and sometimes the presence of pus or discharge. To minimize this risk, surgeons adhere to strict aseptic techniques during surgery, and patients receive postoperative care instructions, including wound hygiene and monitoring for signs of infection [17].

Bleeding or hematoma: Excessive bleeding during surgery or the formation of a hematoma (a blood collection) at the surgical site can occur. Hematomas can lead to discomfort and swelling. If significant, they may require drainage or further intervention. Surgeons take precautions to minimize bleeding during the procedure and closely monitor patients postoperatively [18].

Scarring: Scarring is an inherent outcome of surgical procedures, and the extent of scarring can vary depending on factors such as the surgical approach used and the patient's healing process. Minimally invasive techniques, like endoscopic or laparoscopic approaches, often result in more minor, less noticeable scars. However, traditional open surgery may leave more prominent scars. Surgeons should discuss scarring expectations with patients during pre-operative counseling [19].

Changes in breast sensation or appearance: Surgery, mainly near the areola or nipple, can sometimes lead to changes in breast sensation or appearance. Patients may experience altered nipple sensations or changes in breast shape. Fortunately, these changes are typically mild and temporary. Most patients regain normal breast sensation over time, though it may take several months [20].

Recurrence: While fibroadenoma recurrence after surgical removal is rare, it can occur if not all fibroadenoma tissue is completely excised during the initial procedure. Surgeons aim for complete removal, but there is a small possibility of residual tissue or the development of new fibroadenomas in the same or contralateral breast [21].

Anesthesia risks: General anesthesia, often used for surgical procedures, carries its risks. These risks include complications related to the administration of anesthesia medications, such as allergic reactions, respiratory issues, or adverse reactions to anesthesia drugs. Anesthesiologists closely monitor patients throughout surgery to mitigate these risks [22].

Cosmetic concerns: Patients may have cosmetic concerns related to scarring or changes in breast appearance following surgery. Surgeons should discuss these concerns openly with patients during pre-operative counseling. Clear communication about cosmetic expectations and the potential impact on breast aesthetics can help manage patient expectations and ensure satisfaction with the surgical outcome [23].

Medical management 

The medical management of fibroadenomas involves various strategies tailored to the size, symptoms, and characteristics of the fibroadenomas. In many cases, fibroadenomas may require no active treatment, as they tend to shrink and resolve over time. However, regular monitoring through check-ups and breast examinations is advised to observe changes in size and characteristics. Surgical intervention becomes necessary for giant fibroadenomas or phyllodes tumors, especially if they grow rapidly, cause symptoms, or compress surrounding breast tissues [9]. Surgical excision, the standard procedure, involves cutting out the fibroadenoma, while cryoablation (freezing the fibroadenoma) is a less common alternative. Conservative treatment emphasizes regular follow-ups, typically every six to 12 months, with excision considered if fibroadenomas persist or do not regress by age 35. Achieving hormonal balance, especially in giant fibroadenomas, may lead to shrinkage, favoring conservative management. Close collaboration with healthcare providers is crucial for developing an appropriate management plan, and individuals are encouraged to perform regular breast self-exams and promptly report any new lumps or changes to their healthcare provider [12-14].

Post-operative care

Recovery and Wound Care

Wound care: Proper wound care prevents infection and facilitates healing. Patients should receive clear instructions on how to care for the surgical incision. This typically includes keeping the incision clean and dry. Any dressings or sutures should be managed according to the surgeon's recommendations. Patients should avoid picking at or excessively touching the incision site to reduce the risk of infection [24].

Pain management: Pain and discomfort are expected after surgery. Patients may be prescribed pain medications to manage post-operative pain, or they may be instructed to take over-the-counter pain relievers as directed by their surgeon. The choice of pain management strategy depends on the patient's needs and the extent of discomfort. Patients should clearly understand how to take their prescribed medications and any potential side effects [25].

Activity level: Patients should receive guidance on their activity level during the initial recovery period. Their surgeon recommends limiting strenuous physical activities and heavy lifting for a specified period. However, patients are encouraged to engage in light activities and gentle movements to promote circulation and prevent stiffness. Gradual resumption of normal activities should be discussed and individualized based on the patient's progress [26].

Diet and hydration: Proper nutrition and hydration are crucial for healing. Patients should follow any dietary recommendations provided by their healthcare provider. Maintaining a balanced diet with adequate nutrients and staying hydrated supports the body's ability to heal and recover efficiently [27].

Scarring: Patients should be informed that scarring is a normal healing process. While the appearance of scars can vary from person to person, they typically fade and become less noticeable over time. Patients should be reassured that their surgical team is attentive to minimizing scarring during the procedure and that any concerns can be discussed during follow-up visits [28].

Follow-up instructions: Patients should receive clear instructions regarding when to schedule a follow-up visit with their surgeon. The follow-up visit allows the surgeon to assess the surgical site, remove any sutures or dressings if necessary, and address any concerns or questions the patient may have. It is an opportunity for the patient and surgeon to discuss the healing progress and ensure that the patient is on track for a successful recovery [29].

Pathological Examination of Excised Tissue

Histological evaluation: A pathologist with expertise in breast pathology meticulously examines the excised tissue under a microscope. This histological evaluation is critical in confirming that the tissue is consistent with a fibroadenoma. The pathologist assesses various characteristics of the tissue, including its cellular composition, architectural features, and overall appearance. This examination helps to rule out any signs of malignancy and provides a definitive diagnosis of fibroadenoma [30].

Margin assessment: In cases where the entire fibroadenoma was not removed, such as during a lumpectomy or partial excision, the pathologist may assess the margins of the excised tissue. Margin assessment is essential to ensure that no cancerous cells are present at the edges of the removed tissue. It helps determine whether the surgical procedure successfully removed all fibroadenoma tissue and whether additional intervention is necessary [31].

Diagnostic accuracy: The pathological examination of excised fibroadenoma tissue provides a definitive diagnosis. It serves as a crucial confirmation of the benign nature of the lesion and eliminates any uncertainties about its characteristics. The examination also provides valuable information about the fibroadenoma's cellular and histological features, which may vary among patients. This detailed diagnostic accuracy guides further patient management decisions and helps healthcare providers tailor appropriate treatment plans [5].

Follow-Up Schedule and Monitoring

First post-operative visit: Patients typically have a follow-up appointment with their surgeon within the first week or two after surgery. During this initial visit, the surgeon assesses the surgical site to ensure proper healing. They may also remove any sutures or dressings and address any immediate post-operative concerns or questions the patient may have. This visit is crucial for monitoring the early stages of recovery [32].

Subsequent follow-up: The frequency and duration of follow-up visits may vary based on the patient's clinical condition and the surgeon's recommendations. Subsequent follow-up visits are scheduled from a few weeks to several months during the first year after surgery. These visits allow the surgeon to monitor the patient's progress, assess the surgical site's healing, and address evolving concerns or symptoms [33].

Long-term monitoring: While fibroadenomas are benign, patients must continue routine breast health monitoring beyond the immediate postoperative period. As their healthcare provider recommends, this long-term monitoring involves ongoing breast health assessments, including clinical breast examinations and mammograms. These screenings are vital for the early detection of any new lesions or changes in breast tissue [12].

Patient education: Patient education is fundamental to long-term fibroadenoma care. Patients should be well-informed about the importance of ongoing breast health monitoring and self-care practices. They should receive guidance on conducting regular breast self-examinations and understanding the significance of clinical breast examinations and mammograms in detecting potential abnormalities or concerns. Patient education empowers individuals to actively participate in their breast health and promptly report any changes or symptoms to their healthcare provider [12].

Clinicopathological correlations

Correlation Between Pre-operative Clinical Findings and Post-operative Pathology

A study assessed the correlation between pre-operative clinical findings and post-operative pathology in breast fibroadenoma. The study analyzed the pre-operative diagnosis and matched it with the histopathologic diagnosis. The results showed that the pre-operative diagnosis matched the histopathologic diagnosis 58% of the time but varied significantly with the category [34]. Another study investigated the correlation between pre-operative diagnosis and post-operative pathology reading in pediatric neck masses. The study found that the pre-operative and post-operative diagnoses matched for each category of diagnoses. The difference in the distribution of correct pre-operative diagnoses between six categories of neck masses was statistically significant. The highest number of cases with the correct pre-operative diagnosis was seen with congenital masses, which were correctly diagnosed in 75% of cases (n=109), followed by benign tumors, which were diagnosed in 73.7% of cases (n=19) [34]. In cases where ultrasound was included in the evaluation, there was a trend toward less frequent correlation with post-operative diagnosis in benign tumors, nodal inflammatory, non-nodal inflammatory, and miscellaneous diagnoses. However, regarding the diagnosis of benign tumors, the ultrasound was associated with a trend toward a higher proportion of correct pre-operative diagnoses [34]. These studies suggest that the correlation between pre-operative clinical findings and post-operative pathology can vary depending on the type of tumor and the imaging techniques used in the diagnosis process. In the case of fibroadenoma of the breast, the pre-operative diagnosis matched the histopathologic diagnosis in 58% of cases, indicating room for improvement in the diagnostic process.

Impact of Pathology Results on Treatment Decisions

The impact of pathology results on treatment decisions for fibroadenoma of the breast is significant in guiding appropriate management. Fibroadenomas are typically benign and may not require treatment, as they often shrink or disappear over time [35,36]. However, the fibroadenoma size, patient age, and preference are essential factors in determining the treatment plan [5,36]. In cases where the fibroadenoma is large, proliferates, or causes symptoms, surgical excision may be necessary [35]. Pathology results can also influence the decision for surgical excision, especially if the biopsy results are concerning or the fibroadenoma is complex [37,38]. Additionally, the correlation between pre-operative clinical findings and post-operative pathology is crucial for distinguishing fibroadenomas from breast cancer and avoiding unnecessary surgical interventions [3,4]. Therefore, pathology results are crucial in determining the appropriate action for managing breast fibroadenoma.

Prognostic Significance of Pathological Features

Prognostic value: A pathological examination of fibroadenoma tissue provides valuable information that can affect a patient's future breast health. While fibroadenomas are benign, some may exhibit unusual cellular changes or atypia within the lesion. While these findings do not indicate malignancy, they may be associated with a slightly increased risk of developing other breast conditions in the future. This information is valuable in tailoring the patient's long-term care plan [36].

Risk assessment: Pathological features within fibroadenomas can be used to stratify patients into risk categories based on their likelihood of experiencing specific outcomes. For example, patients with atypical fibroadenomas or specific histological characteristics may be at a slightly higher risk of fibroadenoma recurrence or the development of other breast conditions. Understanding these risk profiles allows healthcare providers to make informed recommendations for follow-up and monitoring [39].

Patient counseling: The ability to interpret the prognostic significance of pathological features empowers healthcare providers to counsel patients effectively. Patients can be informed about their risk profiles based on the pathology results, allowing for more personalized and targeted discussions about long-term breast health surveillance. This counseling includes educating patients about the importance of ongoing breast health monitoring, including regular clinical breast examinations, mammography, and self-breast exams [40].

Treatment outcomes and complications

Long-term Outcomes of Surgical Management

The long-term outcomes of fibroadenoma excision in adolescent and young adult patients have been studied to understand the impact of surgical management. A study reviewed the medical records of females aged 13-35 who underwent fibroadenoma excision and found that most patients were satisfied with the aesthetic outcome of the excision. However, a small proportion desired reconstructive breast surgery, and some reported postoperative breast asymmetry and pain. Additionally, many patients reported the diagnosis of additional fibroadenomas or recurrence of the mass at the excision site, highlighting the importance of addressing these aspects during counseling for treatment options and postoperative follow-up [41]. Another study reported that at three-month follow-up, 89.5% of patients who underwent minimally invasive surgical management for benign breast lesions reported good to excellent cosmetic results. The study also highlighted that benign lesions such as fibroadenomas may recur after the procedure, with the most common complications being hemorrhage, hematoma, and post-procedural pain. Despite variable procedural efficacy, there was high patient satisfaction with the procedure [42-43]. These findings emphasize the need for comprehensive postoperative follow-up and patient counseling regarding potential long-term outcomes, including the possibility of recurrence and further surgical intervention. Healthcare providers must consider these factors when discussing treatment options and managing the long-term outcomes of fibroadenoma excision in adolescent and young adult patients.

Quality of Life After Treatment

The impact of fibroadenoma treatment on patients' quality of life is an important consideration. Different management approaches should be considered based on individual factors, including patient history, age, preferences, fibroadenoma size, complexity, and growth rate [44]. Surgical excision is one of the primary management options, and studies have reported high patient satisfaction with the aesthetic outcomes of minimally invasive surgical management for benign breast lesions, including fibroadenomas [45,46]. However, it is crucial to recognize that recurrence rates after surgical excision of fibroadenomas can range from less than 1% to around 10% [44].

After the procedure, the recovery period is generally short for a simple lumpectomy, with many women experiencing little pain [47]. However, potential complications such as post-procedural pain, hemorrhage, hematoma, and postoperative breast asymmetry have been reported [45,46]. The impact of different treatment modalities on patients' quality of life, including their satisfaction with breast appearance and postoperative recovery, has been studied [45]. Healthcare providers must consider these factors when discussing treatment options and managing the quality of life of patients undergoing fibroadenoma treatment. Comprehensive postoperative follow-up and patient counseling regarding potential long-term outcomes, including the possibility of recurrence and the need for further surgical intervention, are crucial for addressing the treatment's impact on the patient's quality of life [45,46].

High-intensity focused ultrasound (HIFU) and cryosurgery have emerged as promising noninvasive alternatives for treating breast fibroadenomas. HIFU treatment has demonstrated high technical success rates (up to 93.1%) and garnered substantial patient satisfaction. Particularly effective for noninvasive ablation due to the proximity of fibroadenomas to the skin, ultrasound-guided HIFU allows precise targeting and shorter treatment times. The procedure entails using HIFU to ablate fibroadenomas, inducing fibrosis and volume reduction without impacting surrounding tissue. Clinical studies evaluating the efficacy and safety of HIFU show encouraging preliminary results. Cryosurgery, employing extreme cold to destroy fibroadenomas, is a non-invasive treatment option. HIFU and cryosurgery offer potential advantages over surgical excision, including reduced invasiveness, shorter recovery periods, and high patient satisfaction. While these techniques represent significant advancements, continuous clinical research is essential to assess their long-term efficacy and safety thoroughly [40-42].

Emerging research and future directions

Recent Advancements in Fibroadenoma Research

Genomic and molecular studies: Researchers have embarked on comprehensive genomic and molecular studies of fibroadenomas to uncover their development's underlying genetic alterations and molecular pathways. By examining the genetic mutations and gene expression patterns associated with fibroadenomas, scientists aim to gain insights into the factors driving their formation. This research may also suggest potential therapeutic targets for intervention and treatment strategies [48].

Imaging techniques: Advancements in breast imaging have significantly enhanced the accuracy of diagnosing and characterizing fibroadenomas. MRI and contrast-enhanced ultrasound (CEUS) have emerged as valuable tools in the diagnostic armamentarium. These techniques offer improved visualization of lesion vascularity, tissue characteristics, and boundaries, helping clinicians distinguish fibroadenomas from other breast lesions more effectively. The ability to differentiate between benign and malignant lesions with excellent precision aids treatment decision-making and reduces unnecessary surgical procedures [49].

Biomarker discovery: Researchers are actively working to identify specific biomarkers associated with fibroadenomas. These biomarkers may include genetic, protein, or molecular markers that can be detected through non-invasive tests like blood tests or imaging. The discovery of reliable biomarkers for fibroadenomas holds the potential to streamline the diagnostic process, allowing for more accurate differentiation between benign and malignant breast lesions. This, in turn, could reduce the need for invasive procedures such as biopsies and provide patients with faster and less anxiety-inducing diagnoses [50].

Treatment alternatives: Investigational treatment alternatives to surgery are being explored for selected fibroadenomas. Among these alternatives are RFA and cryoablation, which are minimally invasive procedures aimed at destroying fibroadenomas while preserving surrounding breast tissue. RFA uses heat energy, while cryoablation employs freezing to target and shrink the lesions. These techniques offer the potential for reduced scarring, shorter recovery times, and improved cosmetic outcomes. While these treatments are unsuitable for all fibroadenomas, ongoing research is refining patient selection criteria to optimize their use [51].

Promising Diagnostic and Therapeutic Approaches

Biomarker panels: The development of biomarker panels holds significant promise in improving the accuracy of fibroadenoma diagnosis. Researchers are working on identifying specific biomarkers, including genetic, protein, or molecular markers, unique to fibroadenomas. These panels could be integrated into diagnostic tests, such as blood tests or advanced imaging, allowing for more precise differentiation between fibroadenomas and other breast lesions. This approach has the potential to reduce the need for invasive procedures like biopsies, providing patients with faster and less anxiety-inducing diagnoses [52].

Liquid biopsies: Liquid biopsy techniques garner attention for their potential in diagnosing and monitoring fibroadenomas and other breast conditions. These techniques involve analyzing various components in blood samples, such as circulating tumor DNA (ctDNA) and microRNA. Liquid biopsies offer a minimally invasive and accessible means of obtaining valuable diagnostic information. They could provide real-time insights into the presence, characteristics, and changes in fibroadenomas, allowing for more proactive and personalized management approaches [53].

Focused ultrasound ablation (HIFU): HIFU is emerging as a non-invasive treatment option for fibroadenomas. HIFU technology directs focused ultrasound waves precisely at the target lesion, generating heat to ablate the fibroadenoma without surgery. This approach offers several advantages, including minimal scarring, reduced recovery times, and improved cosmetic outcomes. It is particularly promising for patients who prefer non-surgical interventions or have contraindications for surgery [54].

Targeted therapies: Research into targeted therapies for fibroadenomas aims to develop treatments that selectively inhibit the growth of these benign lesions while preserving normal breast tissue. By gaining a deeper understanding of the molecular pathways and genetic alterations involved in fibroadenoma development, scientists are exploring the potential for targeted interventions. These therapies could offer a more tailored and practical approach to managing fibroadenomas, potentially reducing the need for surgery and minimizing associated risks [5].

Areas for Future Investigation

Long-term outcomes: Conducting comprehensive, long-term studies is essential to assess the outcomes of different treatment approaches for fibroadenomas. This includes evaluating the recurrence rates and potential complications associated with minimally invasive techniques and surgical interventions. Understanding the durability of treatment effects is crucial for optimizing patient management strategies [55].

Risk factors: Identifying and elucidating the various risk factors associated with fibroadenoma development remains an important area of research. Investigating hormonal influences, genetic predisposition, and environmental factors can provide valuable insights into the etiology of fibroadenomas. This knowledge can help identify individuals at higher risk and inform the development of preventive strategies or targeted interventions [54].

Patient-reported outcomes: Recognizing the psychosocial and quality-of-life implications of fibroadenoma diagnosis and treatment is vital. Future research should prioritize assessing patient-reported outcomes, including the emotional and psychological impact of fibroadenoma-related experiences. Understanding how these factors influence patients' well-being and body image can guide supportive care and interventions [12].

Precision medicine: Advancements in precision medicine offer exciting prospects for tailoring treatment strategies to individual patients. Investigating the genetic and molecular profiles of fibroadenomas can enable the development of personalized approaches to management. This includes the potential for targeted therapies that selectively address the unique characteristics of each fibroadenoma, optimizing treatment effectiveness while minimizing side effects [56].

Comprehensive registry data: Establishing and maintaining comprehensive registries of fibroadenoma cases can be invaluable resources for ongoing research. These registries can track trends, treatment outcomes, demographic factors, and long-term follow-up data. By consolidating information from diverse patient populations, registries contribute to a deeper understanding of fibroadenomas and provide a foundation for evidence-based decision-making in clinical practice [12].

## Conclusions

In conclusion, fibroadenomas of the breast represent a common and benign yet clinically significant entity that warrants careful evaluation and management. This comprehensive review has highlighted the importance of accurate diagnosis through clinical examination and advanced imaging techniques and the significance of pathological confirmation to rule out malignancy and assess histological features. Surgical management, when indicated, offers favorable long-term outcomes and symptom relief, with emerging minimally invasive approaches showing promise. However, the key to effective fibroadenoma management lies in multidisciplinary collaboration, where healthcare providers from various specialties collaborate to ensure accurate diagnosis, personalized treatment decisions, and holistic patient care. As research continues to uncover new insights and innovative strategies, the future of fibroadenoma management holds the potential for improved diagnostic accuracy, minimally invasive treatments, and enhanced patient outcomes, reaffirming the importance of staying at the forefront of breast health care.
